# Nursing management of fat embolism syndrome complicating tibial fracture in an adolescent: a case report

**DOI:** 10.3389/fsurg.2026.1919622

**Published:** 2026-07-30

**Authors:** Danqiong Wang, Xianjun Shao, Ying Ying

**Affiliations:** 1 Department of of Intensive Care Unit, Ningbo No.6 Hospital, Ningbo, Zhejiang, China; 2 Department of Nursing, Ningbo No.6 Hospital, Ningbo, Zhejiang, China

**Keywords:** fat embolism syndrome, microcirculation, nursing care, personalized nursing plan, social support

## Abstract

Fat Embolism Syndrome is a dangerous complications, which cause by the lipid particles from fracture site block micro micro-circulation vessels in the vital organs (pulmonary, cardiac and Skin). The adolescents who's bone were during an active period of bone growth. Although FES is rare in adolescents, those with long-bone fractures remain at risk. The developmental, psychological, and social needs of adolescent patients differ from those of adults, necessitating age-specific nursing approaches, and the clinical manifestations, psychological and social responses, and rehabilitation needs of adolescents patients are different with adult patients. Therefore, this report takes the nursing practice of patients with 17-year-old tibial fractures and concurrent FES, which focuses on the early identification within the “24–72 h” time window after trauma and the systematic organ support. Based on the nursing practice of this case we concluded our experience about personalized nursing plan that integrates advanced monitoring technology, developmental psychological and social support, and early rehabilitation intervention can truly improve oxygenation, stabilize the internal environment, and promote dual rehabilitation of both the body and the mind. So, it is also an excellent evidence-based reference for the nursing care of FES in adolescents.

## 1 Introduction

Fat embolism syndrome (FES) is considered a rare but potentially life-threatening complication of long-bone fractures, and the reported prevalence is 1%–11% in those with such fractures (1). FES is currently considered to result from multiple interacting mechanisms rather than a single pathological process. These include mechanical obstruction after bone marrow fat enters the bloodstream, biochemical injury mediated by lipid metabolites, inflammatory cascades, disruption of the endothelial barrier, and coagulation abnormalities (2). After the occurrence of long-bone fractures, there is the invasion of bone marrow fat into the venous blood stream causing hypoxemia and dysfunction of microcirculation in the lungs. Furthermore, the breakdown of fat droplets leads to liberation of free fatty acids, resulting in injury of alveolar cells, endothelial cells, inflammation, coagulation disorders, and multiorgan dysfunction with participation of respiratory, nervous, and skin systems (3).

Teenagers belong to an age group with a higher susceptibility to high energy trauma, including injuries in sporting activities and motor vehicle accidents, and hence form an important subgroup of patients with long bone fractures. Even though FES is relatively uncommon in the adolescent age group, the disease can progress quickly and lead to severe complications like hypoxemia, acute respiratory distress syndrome, and cerebral fat embolism (4, 5). Studies on large epidemics on FES among adolescents are few, and most of the studies reported till date have been carried out through case series and individual case reports in Pediatric Intensive Care Units (PICUs). In a systematic review of 18 cases of pediatric FES, respiratory system involvement and neurological manifestations were seen in 77.8% and 83.3% of the patients, respectively. Mechanical ventilation was needed in 88.9% of the cases, and 22.2% had neurological complications (6). From the above, it is clear that even if there is rarely adolescent FES, the syndrome itself is rather serious upon presentation, thus requiring intensive care and therapy. Severe FES patients usually need an integrated approach to care, including respiration, intensive care, fracture repair, and rehabilitation. Nursing care covers treatment, stabilization, and rehabilitation and thus imposes more challenges for team work and coordination of care. In addition, adolescence is a very important phase when it comes to physical and emotional development. Trauma. Severe trauma, the intensive care environment, and prolonged rehabilitation may further increase psychosocial burden, including anxiety and concerns about disease prognosis, changes in self-image, and interruption of schooling (7).

## 2 Clinical course

### 21 Baseline clinical condition

A 17-year-old male patient was admitted to the emergency room due to trauma of the lumbar region and bilateral legs after being involved in a motor vehicle accident about an hour ago. Radiologic and computed tomography images showed that there is a mid-shaft fracture of the left tibia (Figure 1) and injury with bruising of the right knee joint. Emergency treatment included splinting of the left leg with a plaster splint and wound cleansing of the right knee joint followed by suturing. He was then taken to the pediatric orthopedic ward. After admission, the affected limb was immobilized and elevated, and routine electrocardiographic monitoring and symptomatic supportive treatment were provided.

**Figure 1:**
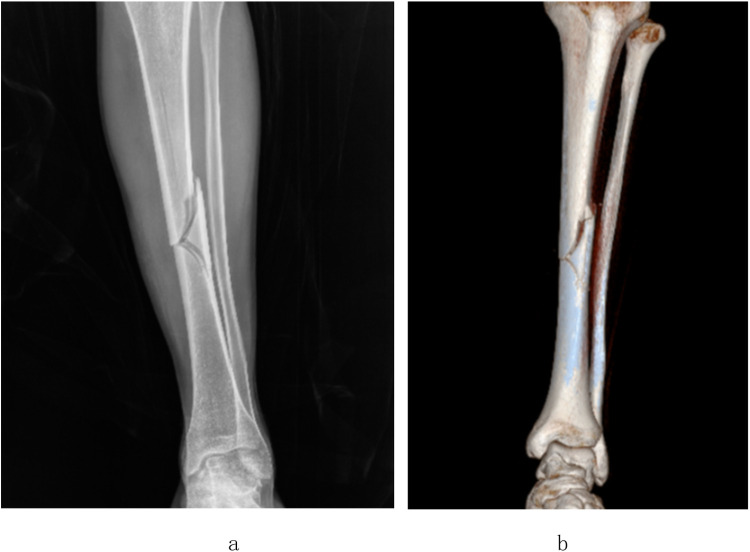
The radiological outcome of this patients, **(a)** the x-ray showed tibial fracture; **(b)** the 3D-reconstructure shown tibial shaft fracture.

Approximately 34 h after injury, the patient experienced a sudden fever of 39.5 °C without any known reason. His percutaneous oxygen saturation (SpO_2_) was 91%. After cooling and oxygen therapy, some improvement was achieved with the fall in body temperature to 38.2 °C and SpO_2_ up to 95%. About 46 h after the injury, the patient experienced an increase in temperature up to 38.6 °C with tachycardia 132 bpm. The patient complained about chest tightness and discomfort and became more restless. Tachypnea (35 breaths per minute) and tachycardia (132 bpm) were noted on physical examination, while blood pressure remained stable. Progressive fall of SpO_2_ from 98% to 70% was noted during oxygen therapy through a nasal cannula at 4 L/min. Auscultation revealed minimal moist rales bilaterally at the base of lungs. Urgent arterial blood gases analysis revealed pH 7.44, PaO_2_ 53 mmHg, and PaCO_2_ 34 mmHg, indicating hypoxemia and respiratory alkalosis. Hematological investigations revealed platelets drop from 152 × 10^9^/L on admission to 104 × 10^9^/L.

The patient presented symptoms of apathy and poor propulsion of his right lower limb 59 h post injury. He had muscle strength of grade II. He also showed signs of transient disorientation and agitation. His Glasgow Coma Scale (GCS) score decreased from 15 to 13 (E4V4M5). A brain magnetic resonance imaging (MRI) done in emergency showed multiple punctate and patchy areas of abnormal signals in bilateral periventricular area, centrum semiovale, and frontoparietal lobe, in line with the “starfield” pattern. The dermatological exam showed multiple pin point petechia in the anterior chest wall, axilla, and the base of the neck that do not blanch upon palpation. By incorporating the trauma history with the classical presentation of the signs, the clinical impression is the patient has a tibial fracture with FES.

### 2.2 Treatment and outcomes

The patient was rapidly transferred to the intensive care unit (ICU) for emergency treatment. The treatment regimen mainly included the following components. First, respiratory support was provided. Conventional oxygen therapy was immediately replaced with high-flow nasal cannula (HFNC) oxygen therapy at a flow rate of 50 L/min, oxygen concentration of 60%, and temperature of 37 °C. The positive airway pressure effect of HFNC was used to promote alveolar expansion, increase the effective ventilatory area, and maintain SpO_2_ above 93%. Second, pharmacological treatment was administered. Based on the patient's body weight of 48 kg and in strict accordance with adolescent dosing standards, methylprednisolone sodium succinate 40 mg was administered intravenously twice daily as prescribed and was subsequently tapered to once daily to reduce the systemic inflammatory response and pulmonary edema. Low-molecular-weight heparin calcium 4,000 units was administered subcutaneously to prevent deep vein thrombosis and further aggravation of microthrombosis. Sivelestat sodium, a protease inhibitor, was also administered by intravenous pump infusion at 0.3 g/day to address the possible inflammatory cascade. Third, organ protection and supportive care were provided. Fluid intake and output were strictly controlled to reduce pulmonary edema. Additional symptomatic supportive measures included neurotrophic therapy, sedation and analgesia, and maintenance of homeostasis. Fourth, two sets of peripheral blood cultures were obtained, and nucleic acid tests for Mycoplasma pneumoniae, SARS-CoV-2, and six respiratory pathogens were performed. All results were negative, further excluding bacterial or viral infection.

After 10 days of comprehensive management and nursing care in the ICU, the patient's dyspnea had markedly improved, consciousness had returned to normal, skin petechiae had completely resolved, arterial blood gas parameters had normalized, muscle strength of the right lower limb had recovered to Grade 5, and pulmonary exudative opacities on chest CT had been significantly absorbed (Figure 2). The patient was then successfully transferred back to the general pediatric orthopedic ward for continued fracture treatment.

**Figure 2:**
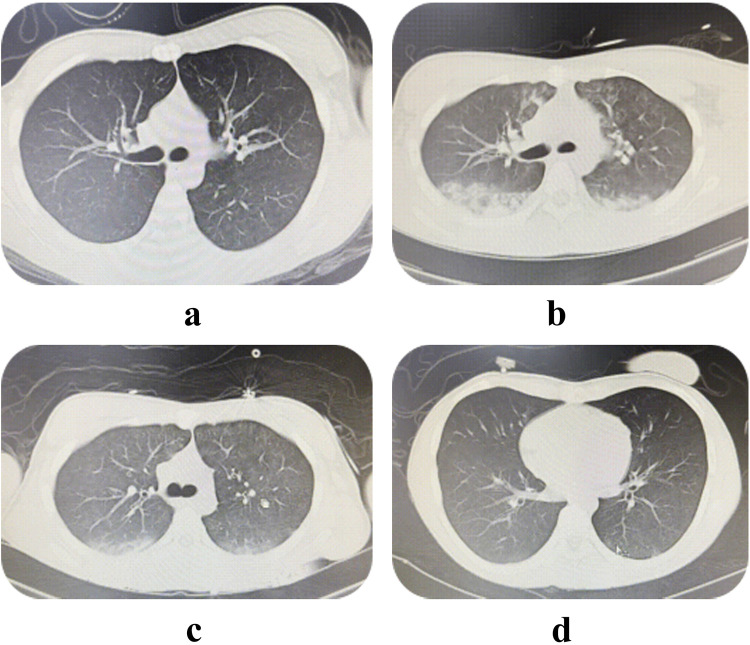
The outcomes of the patient's chest CT during the course of the disease. **(a)** Both sides are expanded, with clear texture, and no obvious abnormal shadows are observed (1 day after injury); **(b)** diffuse increased density shadows in both lungs, with blurred edges (3 days after injury); **(c)** there are diffuse patchy and patchy hyperdense shadows in both lungs, with blurred edges. Compared to the CT scan performed 5 days ago, the exudative changes have significantly improved (8 days after injury). **(d)** There are a few patchy and patchy hyperdense shadows in both lungs, with blurred edges. Compared to the CT scan performed 4 days ago, the exudative changes have significantly improved (12 days after injury).

## 3 Nursing care plan

### 3.1 Sequential management of respiratory support and monitoring

In this case, there was a structured, organized, and purposeful sequential approach to the management of the respiratory system. Initially, the approach included early warning and dynamic evaluation by identifying the first 72 h following the injury as the critical period. The level of nursing care in the pediatric orthopedic ward in this case was level II. Evaluation of the respiratory rate, SpO_2_, and the subjective complaints of the patient were done after 2 h. Chest pain, fever, and tachycardia developed after 46 h of injury, and the nurse rapidly and effectively called for help from the rapid response team of the hospital according to Modified Early Warning Score (MEWS) criteria.

After admission to the ICU, a sequential oxygen therapy approach was adopted through step-wise escalation approach that included nasal cannula oxygen therapy, mask oxygen therapy, and HFNC oxygen therapy. When the patient's SpO_2_ progressively decreased, oxygen therapy was immediately escalated from nasal cannula oxygen to mask oxygen at a flow rate of 10 L/min. SpO_2_ increased from 73% to 89%, but the patient continued to report chest tightness and dyspnea. Arterial blood gas analysis repeated 30 min later showed a PaO_2_ of 53 mmHg. HFNC was therefore initiated, with initial settings of 50 L/min flow and a fraction of inspired oxygen (FiO_2_) of 60%.

### 3.2 Detailed monitoring of neurological symptoms and skin petechiae

The neurological status was tested by pediatric Glasgow Coma Scale (GCS) scale in combination with Richmond Agitation-Sedation Scale (RASS). The assessment was done in a systematic manner every 2 h. Along with standard monitoring of the degree of consciousness, special emphasis was made on changes in the content of consciousness, such as restlessness, apathy, visual hallucinations, and momentary weakness of limbs. Any changes were immediately noted in the nurse's records and informed to the treating doctor.

Evaluation of microcirculation was carried out during the examination. Besides the standard inspection for petechiae on the skin in the chest, neck, axillae, and abdominal regions, the perfusion index (PI) was also assessed using a noninvasive pulse oximetry probe connected to the ECG machine. This method provided an objective criterion for the microcirculatory disturbance and reaction to treatment. Eight days after injury, brain MRI revealed a significant decrease in abnormal signal intensity (Figure 3).

**Figure 3:**
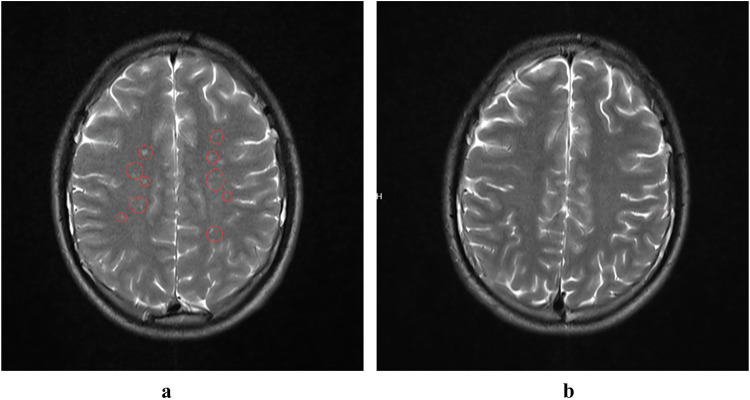
Cranial MRI of the patient. **(a)** The MRI shows multiple punctate or patchy areas of abnormal signal within the brain (3 days after injury). **(b)** The MRI shows no abnormal intracranial signals (8 days after injury).

Skin care and clinical labeling were also emphasized. During each nursing intervention, the patient's whole-body skin condition was examined in a systematic and standardized manner. With the patient's prior consent, photographs of ecchymotic areas were recorded promptly and completely in the work communication group. Changes in the extent, density, and color of the petechiae were actively monitored (Figure 4). Pressure and friction over affected areas were avoided as much as possible. The patient was given clear and patient explanations that these skin findings were part of the disease process. Changes in the extent and color of skin petechiae served as one reference indicator for dynamic assessment of disease progression.

**Figure 4:**
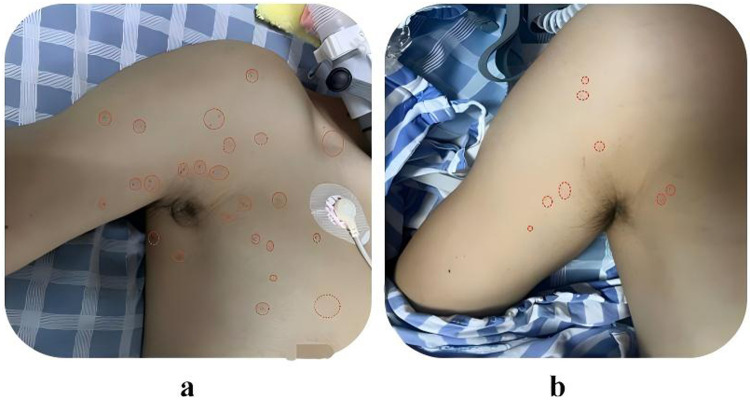
The situation of bleeding spots on the patient's skin during the onset of the disease. **(a)** There are numerous scattered bleeding spots visible on the patient's chest and shoulders (3 days after injure). **(b)** There are no obvious bleeding points on the patient's chest and shoulders (8 days after injure).

### 3.3 Psychosocial support based on adolescent developmental characteristics

Adolescent patients may have marked stress responses to sudden critical illness, the ICU environment, restricted physical activity, and uncertainty about the future. Developmentally appropriate psychological nursing care was therefore provided through a clear, stepwise intervention strategy. First, cognitive guidance was provided. The treatment plan and the functions of monitoring devices were explained to the patient using age-appropriate language to reduce disease-related anxiety. Second, emotional reassurance and empowerment were provided. The patient's feelings of fear and anxiety were first acknowledged through active listening, and his efforts during treatment, such as cooperation with limb function exercises, were clearly affirmed. Third, the family support system was activated. The healthcare team communicated proactively and fully with the parents and guided them to provide emotional support during visits rather than excessive anxiety. A family information board was also established. Disease progression and nursing goals were updated transparently each day to enhance the family's participation in, and perceived control over, the treatment process.

## 4 Discussion

### 4.1 Early recognition and warning value of FES in adolescents

The classic clinical triad of FES consists of respiratory distress, neurological abnormalities, and petechiae, which usually appear 24–72 h after trauma. No specific diagnostic marker is currently available, and diagnosis mainly depends on comprehensive clinical judgment. However, clinical manifestations are not always typical. Early signs may include only decreased oxygen saturation, restlessness, altered consciousness, fever, tachycardia, or imaging abnormalities. These findings can be easily confused with pulmonary infection, traumatic brain injury, pulmonary thromboembolism, or the effects of anesthesia or sedative drugs, leading to missed or delayed diagnosis (8). Nurses therefore play an important role in early disease recognition and clinical warning.

Previous case reports further indicate that the onset and clinical phenotype of FES are markedly heterogeneous. One 16-year-old patient with tibial fracture and pelvic fracture developed severe hypoxemia, impaired consciousness, and acute respiratory distress syndrome within several hours after injury (9). Another patient with a tibial plateau fracture developed delayed, isolated cerebral fat embolism 10 days after surgery (10).

Currently, there are no established criteria recognized as a gold standard for fat embolism syndrome diagnosis. In daily medical practice, the diagnosis of this condition is mainly performed through clinical history, clinical manifestations, and auxiliary investigations results. The criteria that have been formulated by Gurd and Wilson in 1974 are still being used the most often. They divide the clinical symptoms into major and minor. The major criteria include respiratory insufficiency, neurological problems, and petechiae rash. Minor criteria are tachycardia, fever, anemia, thrombocytopenia, increased erythrocyte sedimentation rate, retinal lesions, kidney disorders, and fat globules in the sputum. Normally, the FES can be diagnosed if there are two major criteria present, or one major criterion is associated with four minor criteria (11). Thrombocytopenia, anemia, and hypoxemia are considered the most frequent laboratory abnormalities in FES patients. An arterial blood gas test usually shows low PaO_2_ and early respiratory alkalosis in the patients with FES. Sometimes, the patients may experience hypofibrinogenemia, disorders of coagulation system, hypocalcemia, and hypoalbuminemia. Nevertheless, all these changes are not characteristic of the syndrome only (3).

In this case, the patient had symptoms of fever and lower oxygen saturation without any traditional signs of neurology or petechiae. In case when these symptoms had been evaluated only as inflammation after an injury, it would take much more time to make a correct diagnosis and start appropriate treatment. During all the period of high risk, the nurses monitored the vital parameters of the patient and his oxygenation state. The gradual reduction in oxygen saturation, increase in the respiratory rate, restlessness, and rash were discovered quickly enough.

The patient presented with classic symptoms of FES 46 h following the injury, which is consistent with the time frame at which onset occurs in FES cases. The clinical course showed a somewhat unique presentation. High fever and hypoxemia without any apparent cause became apparent about 34 h following the injury. At 46 h post-injury, there was progressive worsening of oxygenation, alongside chest tightness, difficulty breathing, and tachycardia. By 59 h post-injury, the patient displayed signs of apathy, confusion, weakness in limb movements, and petechiae. During the diagnostic process, the medical team evaluated the patient based on his history of long bone fracture, progressive hypoxemia, neurological dysfunction, and petechiae. Based on the Gurd diagnostic criteria, the patient met several major criteria. There was a significant reduction in the number of platelets from the baseline value. MRI of the brain showed the classic “starfield” appearance. There was no laboratory evidence of infection, either from blood culture, respiratory pathogens test, or other imaging modalities.

In addition to routine vital sign monitoring, the nursing team dynamically assessed respiratory function, neurological status, and tissue perfusion using respiratory rate, SpO_2_, GCS, RASS, MEWS, and PI. Approximately 46 h after injury, the respiratory rate and arterial blood gas results met the reference criteria for respiratory abnormalities proposed by Lindeque et al., including respiratory rate ≥35 breaths/min, PaO_2_ < 8.0 kPa (approximately 60 mmHg), PaCO_2_ < 7.3 kPa (approximately 55 mmHg), and persistent respiratory distress (11). The Glasgow Coma Scale is widely used to monitor neurological involvement in FES. Previous evidence suggests that a GCS score ≤9 and lower GCS scores are associated with poor prognosis in patients with FES (12). MEWS helps identify clinical deterioration and provides a reference for activation of the rapid response team. RASS is used to assess agitation and changes in consciousness. Although the use of PI in FES is not yet supported by sufficient evidence, its dynamic changes may partly reflect systemic perfusion status and provide additional information for clinical monitoring.

### 4.2 Systematic nursing care centered on respiratory support

Respiratory impairment is the main manifestation of organ dysfunction in FES. Some patients may progress to acute respiratory distress syndrome and require mechanical ventilation. Severe hypoxemia is a key factor affecting prognosis (3).

Given the progressive decline of SpO_2_ in the patient, nurses were able to observe an increased sensation of chest tightness along with dyspnea. A prompt notification was made to the doctor who then increased the oxygen flow rate to 10 L/min using a face mask. Despite the increase in SpO_2_ from 73% to 89%, the patient still had dyspnea. Further assessment through arterial blood gas showed a PaO_2_ of 53 mmHg, suggesting that there was no much improvement in oxygenation. In light of this, high flow nasal oxygen therapy was initiated.

HFNC can improve oxygenation by providing a stable FiO_2_, reducing dead space, reducing work of breathing and having a positive end expiratory pressure effect. There are many benefits of using HFNC oxygen therapy compared to other forms of oxygen therapy and it is comparable to non-invasive ventilation in hypoxemic respiratory failure without being inferior to the latter (13).

In this case, progressive hypoxemia was observed in the patient throughout the illness period. Dynamic evaluation of the oxygenation state of the patient and the work of breathing load enabled nurses to help doctors begin HFNC therapy for the patient. The initial parameters that were established included 50 L/min flow and 60% FiO_2_. Treatment parameters were adjusted based on the patient's oxygenation state to ensure that SpO_2_ is maintained at more than 92% (14). In the process of treatment, the main criteria for the patient's monitoring involved respiratory rate, SpO_2_, blood gas analysis, and dyspnea of the patient. Moreover, airway humidification, nasal and facial skin protection, and comfort of the patient were considerably improved. As a result of treatment with HFNC, oxygenation improved progressively, dyspnea was significantly reduced, and the patient was protected from endotracheal intubation and invasive mechanical ventilation.

### 4.3 Multidisciplinary collaboration and supportive treatment

Regarding pharmacological treatment, glucocorticoids are mainly used to prevent the inflammation from getting worse and to reduce the elevated permeability of the pulmonary capillaries. In some cases where patients are critically ill, improvement of hemodynamic function may be rapid following the use of methylprednisolone (15). During nursing care, blood glucose fluctuations, blood pressure changes, water and sodium retention, and infection risk should be closely monitored (16). Anticoagulation is used to prevent secondary microthrombosis, but bleeding risk must be dynamically assessed. Nurses have key monitoring responsibilities during these treatments, including monitoring vital signs, observing bleeding tendencies, and dynamically tracking laboratory indicators.

The core of neurological nursing is early identification of neurological impairment and dynamic assessment of its progression. At present, recovery from cerebral FES is mainly evaluated using GCS scores and imaging findings. However, the degree of radiological lesion absorption is not always synchronized with neurological functional recovery (17, 18).

### 4.4 Whole-course nursing model based on adolescent physical and psychological characteristics

The nursing practice in this case emphasized the physical and psychological developmental characteristics of adolescents and their need for long-term functional recovery. It reflected a patient-centered holistic nursing approach in several aspects.

First, medication-related nursing care was strengthened. During glucocorticoid therapy, adverse effects such as acne and hirsutism may occur. These appearance-related changes may have a greater impact on quality of life and psychological health in adolescents than in adults (19). Health education and psychological support were provided to help the patient correctly understand medication-related reactions, reduce anxiety and inferiority, and improve treatment adherence. This reflected the integration of physical and psychological care.

Second, psychosocial support was provided. Health education and health promotion are important components of modern nursing practice (20). In adolescence, complex illness may trigger a sense of hopelessness, which may manifest as avoidance behavior, uncertainty or negative expectations about the future, and inability to make future plans. Nursing measures such as showing concern, attention, and interest, providing truthful explanations, helping adolescents recognize small achievements, and sharing information about peers with similar experiences may promote willingness for self-support and thereby improve disease prognosis (21).

Third, rehabilitation intervention was initiated. Rehabilitation should begin as early as possible, with the timing determined according to the patient's injury and physical function. The main goals of acute-phase rehabilitation include improving rehabilitation potential and preventing complications such as thromboembolic events and pulmonary complications (22). For critically ill patients, early mobilization within 72 h of ICU admission has been shown to reduce ICU-acquired weakness and improve functional outcomes (23). In this case, rehabilitation began during the ICU stage. From the acute ICU phase, attention was paid to maintaining the overall condition, promoting respiratory function, and preventing bed rest-related complications. During the stable phase, rehabilitation gradually transitioned to functional training of the affected limb. This formed a continuous rehabilitation pathway consisting of safe immobilization, systemic maintenance, local functional recovery, and engagement-based adherence management. This approach is also consistent with recent concepts emphasizing patient empowerment, hope promotion, and cognitive-behavioral rehabilitation (24).

### 4.5 Limitations and future perspectives

Current studies on FES in China and other countries mainly focus on acute diagnosis and supportive treatment. Rehabilitation studies addressing post-discharge neurocognitive recovery, motor function reconstruction, and psychosocial adaptation remain limited. This case presents practical experience in systematic treatment and nursing care for an adolescent with severe FES under a multidisciplinary collaboration model. However, acute-phase pain management could be further optimized. The goals of pain management are not only to relieve pain but also to promote functional recovery and improve treatment adherence. Standardized pain assessment and multimodal analgesia should also be emphasized (25).

In the future, more standardized and quantitative pain assessment tools may be introduced in addition to routine pain monitoring. These tools could be used to dynamically assess pain severity and its effects on the patient's physiological and psychological status. The value of multimodal analgesic strategies in adolescents with FES should also be explored, including standardized use of nonsteroidal anti-inflammatory drugs and nonpharmacological interventions such as virtual reality-based distraction. These strategies may improve patient comfort, treatment adherence, and participation in rehabilitation.

In addition, a follow-up study of patients with cerebral FES showed that although most patients survived to discharge, 57% of survivors had mild to moderate functional impairment (26). This finding suggests that a long-term follow-up mechanism should be established for adolescents with major trauma and related complications. Long-term outcomes, including neurocognitive function, mental health, quality of life, learning ability, and social adaptation, should be systematically evaluated. Such evidence may support the development of a whole-course nursing model that extends from acute treatment to long-term rehabilitation.

## Data Availability

The original contributions presented in the study are included in the article/Supplementary Material, further inquiries can be directed to the corresponding author.
